# Rural Doctors’ Views on and Experiences with Evidence-Based Medicine: The FrEEDoM Qualitative Study

**DOI:** 10.1371/journal.pone.0152649

**Published:** 2016-03-31

**Authors:** Ranita Hisham, Su May Liew, Chirk Jenn Ng, Kamaliah Mohd Nor, Iskandar Firzada Osman, Gah Juan Ho, Nurazira Hamzah, Paul Glasziou

**Affiliations:** 1 Department of Primary Care Medicine, Faculty of Medicine, University of Malaya, Kuala Lumpur, Malaysia; 2 Family Health Development Division, Ministry of Health, Putrajaya, Malaysia; 3 Klinik Kesihatan Jaya Gading, Kuantan, Pahang, Malaysia; 4 Centre of Research in Evidence-Based Practice, Faculty of Health Sciences and Medicine, Bond University, Queensland, Australia; University Hospital Lausanne, SWITZERLAND

## Abstract

**Background:**

Evidence-based medicine is the integration of individual clinical expertise, best external evidence and patient values which was introduced more than two decades ago. Yet, primary care physicians in Malaysia face unique barriers in accessing scientific literature and applying it to their clinical practice.

**Aim:**

This study aimed to explore the views and experiences of rural doctors’ about evidence-based medicine in their daily clinical practice in a rural primary care setting.

**Methods:**

Qualitative methodology was used. The interviews were conducted in June 2013 in two rural health clinics in Malaysia. The participants were recruited using purposive sampling. Four focus group discussions with 15 medical officers and three individual in-depth interviews with family medicine specialists were carried out. All interviews were conducted using a topic guide and were audio-recorded, transcribed verbatim, checked and analyzed using a thematic approach.

**Results:**

Key themes identified were: (1) doctors viewed evidence-based medicine mainly as statistics, research and guidelines, (2) reactions to evidence-based medicine were largely negative, (3) doctors relied on specialists, peers, guidelines and non-evidence based internet sources for information, (4) information sources were accessed using novel methods such as mobile applications and (5) there are several barriers to evidence-based practice, including doctor-, evidence-based medicine-, patient- and system-related factors. These included inadequacies in knowledge, attitude, management support, time and access to evidence-based information sources. Participants recommended the use of online services to support evidence-based practice in the rural settings.

**Conclusion:**

The level of evidence-based practice is low in the rural setting due to poor awareness, knowledge, attitude and resources. Doctors use non-evidence based sources and access them through new methods such as messaging applications. Further research is recommended to develop and evaluate interventions to overcome the identified barriers.

## Introduction

Evidence-based medicine (EBM) is the “integration of best research evidence with clinical expertise and patient values” [1]. It is the assumption of most patients that modern medicine is based on evidence from good medical research [2]. Yet, it has been reported that more than one-third of patients do not receive evidence-based health care and as many as a quarter receive harmful or unnecessary care [3]. A study has found that EBM reduces mortality and shortens hospital stays [4]. However, incorporating EBM into clinical practice remains challenging [5]. A systematic review identified many barriers to the implementation and practice of EBM including lack of knowledge, resources, time and skills [6]. Doctors feel overloaded with information, but find themselves still unable to answer clinical questions with evidence. In one study, reasons provided by primary care clinicians for not using clinical evidence were lack of time, distrust of the information given and a perception that the evidence is not applicable to their practice [7]. While making clinical decisions, doctors preferred to rely on clinical experience, colleagues’ opinions and electronic information resources rather than referring directly to EBM literature [8].

In rural areas, the concentration of poverty, low health status and high burden of disease create a need for greater attention to healthcare provision [9]. Access to healthcare is limited in rural areas. Also, these settings are usually geographically remote, making it harder for physicians to attend continuing medical education activities [10,11]. EBM may have particular relevance and advantages for rural health practice. In Australia, the level of clinical knowledge was found to be low among rural practitioners. The evidence-based approach is imperative in a setting where resources are limited because this approach emphasizes the use of treatments with proven efficacy. The practice of EBM enables health service managers to determine services that will give the greatest benefit to the community served [12,13].

However, there are challenges in adopting EBM in rural practice. These include greater time pressure [14], inapplicable standard clinical guidelines, poorer access to research databases, isolation and lack of organizational support and resources [15,16]. Rural practitioners also tend to be generalists [17,18]; their breadth of practice makes implementation of EBM challenging. There has been little empirical research on the processes that drive the adoption of EBM in this setting [11].

This study is part of a larger project, the Frontline Equitable Evidence-based Decision-Making study (FrEEDoM), which aims to develop an intervention to assist doctors in retrieving evidence to assist clinical decision making. The first phase of this project is to determine the views and experiences of the doctors in a rural healthcare setting regarding EBM. We aimed to explore their attitudes, barriers and needs with regards to the incorporation of clinical evidence into daily practice.

## Method

### Study Design and Setting

A qualitative methodology was chosen to allow the exploration of participants’ personal views and experiences. Semi structured in-depth interviews (IDIs) and focus group discussions (FGDs) were used. Participants were family medicine specialists (FMS) who are doctors with postgraduate qualifications and MO who are doctors without postgraduate qualifications. We interviewed the FMSs individually because medical officers who worked under them may have felt inhibited in the discussion if they were in the same focus group.

The interviews were conducted in June 2013 in two rural districts (Maran and Bentong) in Pahang—the largest state in Peninsular Malaysia. Rural was defined as areas with a population of less than 10,000 people with agriculture and natural resources [19] or areas with a population density of less than 400 people per square kilometer [20]. Maran has a population density of 59 residents per square kilometer [19]. There are seven community clinics and the nearest hospital is 44 km from the main clinic—Maran Health Clinic. The population density of Bentong is 52 residents per square kilometer [19]. There are seven community clinics and one hospital in this district. Many of the residents in these two districts work in palm oil plantations.

### Ethics

We obtained ethical approval for the study from the National Medical Research Register—Medical Research Ethics Committee (MREC reference: NMRR ID NMRR-12-1262-14539 S2 R0). All participants received RM100 (USD 30) as reimbursement for their time and travel expenses.

### Participant Recruitment

Maran and Bentong District Health Offices provided contact details of the doctors serving at the district clinics. All 46 doctors working in public healthcare clinics in the two districts were invited via emails and phone calls to participate in the study. Of these, 18 participants from seven clinics (Maran n = 9, Bentong n = 9) were recruited. Four FGDs with 15 medical officers and three individual in-depth interviews with FMSs were carried out. The interviews were conducted in two rural health clinics which was located at Karak and Maran Health Clinics by experienced facilitators (NCJ and LSM). Both are academic primary care physicians and advocates of EBM. All participants received and went through the participant information sheet before giving written consent. They were also assured of anonymity and confidentiality of the data. The demographic and clinical backgrounds of the participants are presented in Table 1.

**Table 1:** Participants’ demographic and clinical background.

Characteristics	Number of participants (n = 18)
Gender	
Male	6
Female	12
Position	
Family Medicine Specialist	3
Medical Officer	15
Mean of years of practice, SD [range]	6.2, SD 6.3 [3–28]
Ever attended EBM training course	
Yes	2
No	16
Access to computer/internet	18
Medium of search for medical information*	
Clinic computers	13
Hand phone	14
Tablets	3
Personal laptop	1
Clinical Practice Guidelines hard copy	1

*Multiple answers were allowed

### Interview Questions

**Fig 1:**
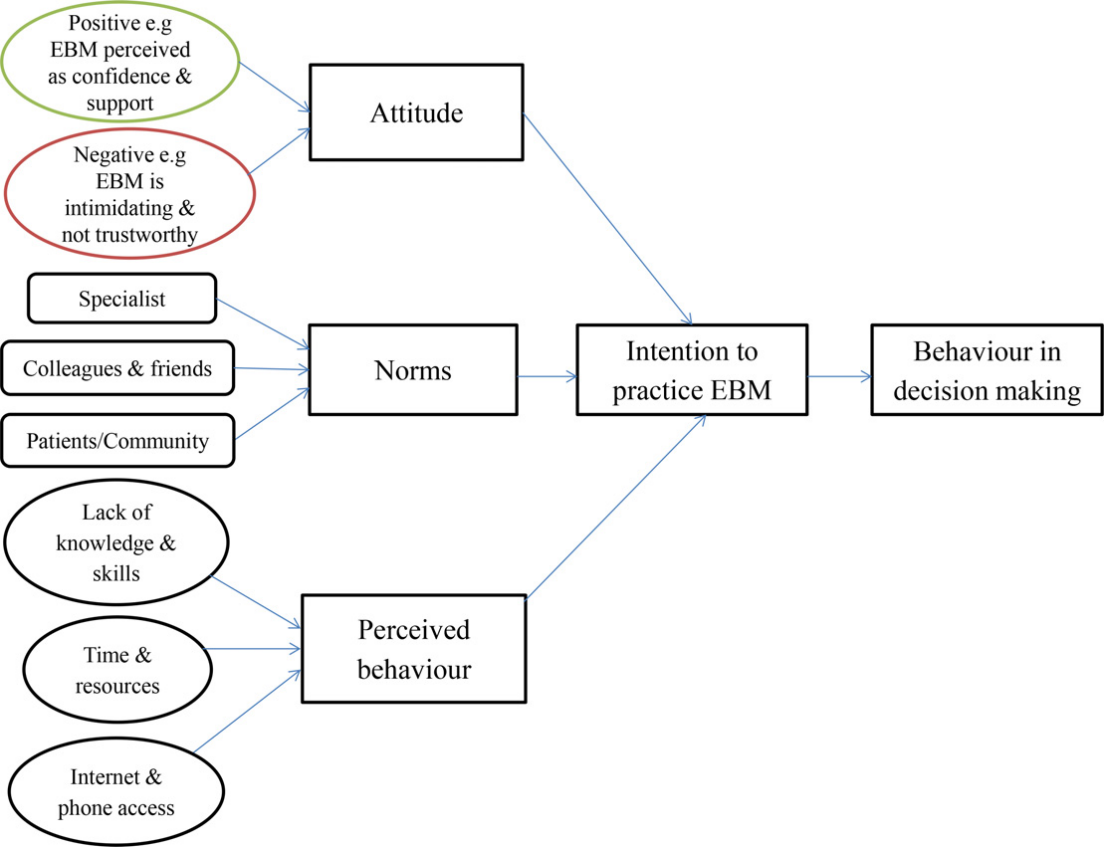
Theory of Planned Behavior (revised).

The interview questions were developed based on the theory of planned behaviour (TPB), which is an extensively used psychological model for understanding human behavior [21]. It infers that people are far more likely to behave in a specific way, if they form a conscious intention to do so and this intention is the major determinant of whether the behavior will happen. TPB consists of three factors that will influence an individual’s behavior, attitude, norms and perceived behaviour. The framework as shown in Fig 1 theorises that the formulation of the intention to practice EBM is derived from the combination of three key factors: attitudes toward the use of EBM, the extent to which the doctors perceived subjective pressure to incorporate EBM into treatment decisions and doctors’ perceived capabilities of practicing EBM. This framework and findings from other studies in the medical literature were used to formulate questions for the topic guide. Interviews were conducted in English since most doctors use English professionally. The topic guide is available in S1 Appendix.

### Data Analysis

All the interviews were audio-recorded, transcribed verbatim and checked. The qualitative research software, NVivo10 was used to help sort, arrange and classify data. These data were analyzed using thematic analysis. Coding was done by a researcher with a nursing degree (NAH), a researcher with a degree in biomedical sciences (HGJ) and a health sciences librarian (RH). The data were read several times for familiarization and immersion. Text was labeled line by line to form the nodes. Nodes with similar themes were grouped to form categories. The coding was checked independently by LSM and NCJ, and discrepancies were resolved via discussion and consensus.The rest of the transcript was coded using the framework. Field notes were used to triangulate the data. Themes were then refined by removing or recoding redundant and infrequent nodes. These were then rechecked by LSM and NCJ to produce the final themes.

## Results

We identified three themes in the context of practicing EBM in the rural setting: (1) doctors’ views and experiences in practicing EBM, (2) their barriers to incorporating EBM in the daily practice, and (3) their needs in order to facilitate evidence-based practice.

### Doctors’ Views and Experiences in Practicing EBM

#### Doctors’ Definition of EBM

Doctors associated EBM with statistics, clinical practice guidelines (CPGs) and evidence from research. It was believed that research and clinical trials produced the best evidence that can then be trusted and followed.

“Evidence means that when you want to treat a patient, you have to make sure that the management that you give actually depends on research data. But CPGs are also research-based. Somehow it’s just more reliable, available or acceptable in our community to be used.” (P2 FGD 1, 28-year-old woman)

#### Importance of EBM

The participants expressed mixed attitudes toward EBM. Some felt that EBM was helpful in clinical decision making. Doctors felt that the EBM is important because good research evidence was seen to be universal; they can access the same source and share knowledge with doctors elsewhere.

“I treat it [EBM] as the definite goal. This is what we really want for our patient, what we can offer as a substitute.” (P5 FGD 3, 28-year-old man)

“My opinion is, I think it’s [EBM] really good, I mean, if there is an evidence-based resources then I will be more confident using it because it makes a lot of sense. Because it’s based on the evidence so I find it rather useful.” (P8 FGD 1, 31-year-old man)

However, not all participants had the same opinion; some felt that EBM was not important or necessary to clinical practice. Many felt frustrated because they were not taught about EBM in depth during medical training. They were negative about the practice of EBM in their daily work. EBM was perceived as requiring the use of a lot of resources such as time, reading and skills.

“Actually the first thing that I know about EBM is you have to read a lot. You have to spend more time and be more passionate to read journals in order to be knowledgeable, well…it’s just that we are not really a reading nation…”(P2-01, 28-year-old woman)

“Because we don't understand what is it [EBM] and then when we have the interest, of course we will go through; if we have no interest, we try to skip.” (P5 FGD 1, 33-year-old man)

One doctor stated that EBM came second, after clinical experience. With a background in public health, he perceived that research outcomes and statistics are easily manipulated.

“Certain things you have to follow the evidence…let’s say if you don’t have knowledge about the management, then it’s better to follow evidence-based. But sometimes certain things you already know the practice better, then it’s fine. I think the evidence comes second.”(P1 IDI 1, 55-year-old man)

#### Doctors’ Experiences in Practicing EBM

During the interviews, participants were probed on the practice of EBM in their daily clinical routine. The participants spoke about their actual use of a mobile application which allows them to broadcast their clinical question in real time to a group of their peers. The mobile application, WhatsApp, was used by the participants because it allowed immediate response from their peers. Their peer group would comprise colleagues in different specialties; those considered ‘experts’ such as a medical officer working in a hospital will be able to give answers based on their current practice. This was a form of ‘peer-to-peer’ information sharing that took place over an electronic online resource. This information exchange, though not secure, is usually limited to a selected group of participants and is not accessible to the public.

“We have a good discussion group, so we use our WhatsApp usually for our discussion such as picture sharing. It’s easy to discuss, for example we can just snap a photo of the X-ray of some fractures that we are not sure of the management and send it via WhatsApp. Sometimes if you ask a silly question also they will answer because we are friends.” (P5 FGD 1, 33-year-old man)

“WhatsApp is the best thing ever designed I guess! So usually when we call them to refer anything, the MOs will ask us to send the picture, you know last time we used to use MMS (Multimedia Messaging Service), but I guess it took some time and then sometimes we have a problem with it but WhatsApp is one of the easiest thing.” (P7 FGD 1, 31-year-old woman

#### Sources of Clinical Evidence Used by Doctors

The doctors’ use of clinical information sources for the purpose of clinical decision making differed according to accessibility. Due to the lack of resources and facilities in their settings, doctors tended to refer to quick and easily accessible resources such as medical information websites or their specialists. The information sources seem to be peers, CPGs, specialists, and internet sources such as Medscape.

#### Specialists

Many participants relied on the family medicine or the hospital specialists to make the decision. They felt that this approach was more reliable for emergency cases and when they were uncertain of information retrieved from other sources. This approach was also perceived to protect them from potential litigations.

“The main person is the specialist, we need to call them even though we know the answer will be from other sources, first is we need to tell right there ok, discuss with whom, spoken to whom because later if medico legal they [Specialist] will be the one who are going to back us up.” (P5 FGD 1, 33-year-old man)

“We discuss with our specialist. As I told you they’re not the main concern, but the main priority is that the pediatrics and antenatal. This two is like a national mortality. So when it comes to these two, we just called up the specialist and then just discuss with them.” (P5 FGD 1, 33-year-old man)

#### Guidelines

Participants still relied on CPGs (Clinical Practice Guidelines) as a source of information on managing their patients. Many felt that these recommendations published by the Ministry of Heath, Malaysia were based on well-appraised evidence and should be adhered to. Doctors also mentioned international guidelines as a source of information, e.g., NICE guidelines.

“Other people have made it [CPG] easy for us to implement evidence-based medicine.” (P4 FGD 1, 29-year-old woman)

“We can depend on solid facts based on this CPG and we can always use it. We feel safe to follow this and if something happened to the patient based on the management that we’ve done, that means you are not in trouble you see? Rather than, oh like I say we have the same situation and Dr F will go with the EBM, I’ll go with the CPG, I think I will I can sleep better at night. I think I can sleep better rather than Dr F, it’s I think how it works here. How people take things. (P2 FGD 2, 29-year-old woman)

Some of the doctors shared their experiences in using EBM in daily clinical practice. Apparently, most of the doctors were relying on pre-appraised evidence, which were mainly the guidelines to seek quick answers on patient management.

“Usually we use Clinical Practice Guidelines or the evidence-based guidelines as our guide. I will take pictures or jot down the management and if I encounter this kind of cases, then I will know what to do first. You should look for this [management] then after that you think whether the management of the disease is possible. This is how I practice, but usually you need time to go and read up.” (P8 FGD 1, 31-year-old man)

#### Other sources of information

Participants also consulted friends and colleagues to get immediate response and solutions to clinical problems.

“Because if we are not sure then we will discuss among ourselves and then if still can’t get answers, then we will ask our boss and the specialists.” (P8 FGD 2, 29-year-old man)

“We have friends and colleagues’ who work in hospitals, we share what we do here in primary care and how is their management in tertiary settings. They also have their specialist to consult…” (P2 FGD 2, 29-year-woman)

Participants generally considered the internet as one of the more important sources of information. The websites that they frequently used were Wikipedia, Medscape, WebMD and e-Medicine. Only a handful of doctors mentioned medical databases such as PubMed and UpToDate. “Googling” is the preferred means to search for information.

“Usually I would search for the answers through internet or maybe I find the information from Google.com. I will search whatever I want to find. I will choose reliable websites such as e-medicine or some journal.” (P8 FGD 1, 31-year-old man)

“It depends on what type of clinical questions, usually I will just refer to the CPG, if there’s no CPG, I will usually go to Medscape” (P5 IDI, 36-year-old woman)

### Barriers to the Practice of EBM

The barriers that emerged can be categorized into doctor, EBM, patient and system barriers.

#### Doctors’ barrier

Participants felt that they faced a lot of difficulties in practicing EBM. The main problem was lack of time, limited exposure to EBM and skills. Doctors also struggled to implement EBM in their daily practice because it required considerable effort. Training was limited in medical schools. Most of the training provided focused on statistical knowledge rather than teaching students on how to apply evidence to patient care in the ‘real world.’

“We only had little exposure to it [EBM]. Because we just studied for the examination purpose and they just gave us a project and you are required to sum it up, do all the confidence interval and all those kind of thing, just one project about it.” (P8 FGD 1, 31-year-old man)

#### EBM-related barriers

This barrier was mainly due to the perceived image of EBM itself. The term of EBM was felt to be intimidating. One participant perceived EBM to be a measure used by health authorities to check on his work quality.

… evidence-based …maybe they want to ask me questions what’s happening… like an audit. (P5 FGD 1, 33-year-old man)

“EBM sounds like an assessment of our knowledge… whether you are good doctors or not. Very scared…” (P2 FGD 1, 28-year-old woman)

Evidence was also seen to be unreliable because it changed over time.

“EBM was also too volatile; mobile too. I don't know I can use it …dynamic you know? Ever changing …regularly comes up with new [evidence]…” (P5 FGD 3, 28-year-old man)

EBM was also seen as being irrelevant to rural settings. Participants also made the observation that researches were mainly conducted in urban settings and felt that results cannot be translated to rural practice.

“To me sometimes evidence-based medicine cannot [be] applied to our local situation, maybe most of the research done at urban area settings so they have their own guidelines, less research conducted in Pahang.” (P6 FGD 1, 32-year-old woman)

#### Patients’ barriers

The participants found it difficult to practice EBM when their patients preferred to seek alternative treatment, self-medicate or self-prescribe for their illnesses. The doctors felt helpless as these factors are perceived to be out of their control.

“In FELDA [Malaysian rural land development scheme] areas, most of the patients are peneroka [settlers], so most of them take the traditional medicine. Problem konon [as though] patient cannot agree with our medicine (P6 FGD 1, 32-year-old woman)

“Like those orang asli [aborigines] they have their own belief, their rituals and everything, they say ‘Oh I don't want to go and see any doctor, I already did jampi [spell], I already went to see bomoh [shaman] …my kid inside is protected, they don't want to do their antenatal care’…” (P1 FGD 2, 28-year-old woman) …”

Patients tended to ignore medical advice, even though it might be evidence-based. Participants felt that the community was not aware of EBM and refused recommended practice, making it harder for doctors to also practice EBM.

“Another main issue is patients self-medication. They can walk in and buy anything they want from the pharmacies, the higher end antibiotic to lower end antibiotic, anything there is. Can go walk into a pharmacy and then patient only come to us when they already have high fever. They already have taken their own medication- antibiotic, they go to the GP clinic, then after 2 days jump to another GP! Without …they don't have any blood taking. I’m doing locum also. GP like me also sometimes I never give medicine that the patient wants, they will jump to another clinic. So, to avoid that to happen, I just give you my medication. After two to three times go to the GPs, then they will come to KK [Klinik Kesihatan (Health care clinic)], by that time conditions already worsen.” (P5 FGD 1, 33-year-old man)

“I think rather than just educating the doctors, we should educate the community too. Like for example, drugs are available in the shop [pharmacies] and you advise people don't take but we are selling it. System! Yes, there is a problem with the system! “(P4 FGD 1, 29-year-old woman)

#### System barriers

There were several factors arising from the healthcare system and policies that impede the practice of EBM in the rural primary care setting, and they include heavy workload, lack of resources, difficulty in accessing information and variations in clinical standards and practice.

Time constraint and heavy patient load were cited as major hindrances in practicing EBM.

“It’s not like one person we are taking care of, the ratio here is crazy for Mempaga and Karak and sometimes it’s really crazy! 1 to 100 and all the cases are complicated you know.” (P2 FGD 2, 29-year-old woman)

“It’s difficult sometimes to practice based on all this research, evidence and all, when we have to search then it is going to be quite difficult…[Laughed] because we have lots of patients lining up outside. It’s going to take some time and just because people like to complain.” (P6 FGD 2, 31-year-old man)

The health clinics lacked facilities and resources that were seen as essential for the doctors to practice EBM. For instance, there was a limited choice of medications available in the rural health clinics.

“We are in KK [health care clinics], especially KK we don't have much medicine. Then, we admit patients. Our resources are very limited. So we cannot admit the patient and we cannot send the patient back home also because when we send the patient back home, we don't know what's going to happen at home. Whether they are going to follow what we say or whether they take their medication or not, we don't know. So, the best is, we call up the specialist, talk to them and send them to the hospital.” (P5 FGD 1, 33-year-old man)

“But usually we won’t be able [to practice EBM] because we have so limited resources back in clinics. Mmm because based on evidence of course the best medication would be this, this or this and yet we have limited drugs and we only have this; by hook or by crook we have to manage our patients with the resources that you have.” (P2 FGD 2, 29-year-old woman)

The doctors also had difficulty in accessing the information in their rural practice. The internet connection was unreliable and the doctors mostly relied on their personal internet service provider using their mobile phones. Accessing the full-text publication was also difficult because the organization did not subscribe to these databases.

“Connection is quite a problem.” (P2 FGD 1, 28-year-old woman)

“Soft copy yes, but hard copy I rarely use it now and then it [journal] is expensive to get and sometimes they just give the summary. We cannot download and if need to buy it, I don’t subscribe to it.” (P9 IDI 1, 43-year-old woman)

### Recommendations for Improving EBM Practice

Most of the participants wanted to have a more reliable internet connection to search for information and practice EBM. Without that, it would seem useless for them to consider interventions to improve the practice of EBM.

“I think with better internet access and proper specific websites for us, you know some websites that we can go look specifically for this kind of thing.” (P7 FGD 1, 31-year-old woman)

One of the FMSs felt that teleconferencing would be helpful. She had experienced journal club by teleconferencing as a medical student during her rural posting abroad. It allowed the sharing of information and continuing medical education with colleagues in remote settings.

“It would be nice if you have the teleconference. Everybody will actually receive it. Ok, so that might work better. The Skype will solve the transportation problems.I find it quite useful.” (P5 IDI, 36-year-old woman)

Some doctors wanted to share information publicly through forums or via Facebook.

“Yes! I think if you were to try via Facebook, it would be the best….we will get notification for the answers given right.” (P4 FGD 1, 29-year-old woman)

“Because for example, nowadays everybody has Facebook account, even our Prime Minister has a Facebook account to communicate with people right? So for us to practice EBM sometimes we get the experience by making friends with doctors, senior doctors and consultants through Facebook, sometimes they give quite useful information.” (P7 FGD 1, 31 years old woman)

Some requested an all day telephone helpline service to support doctors in decision making. Participants wanted immediate answers, especially with regards to emergency cases.

#### EBM Support Services

Doctors were asked for suggestions on the development of an EBM support service. They recommended the use of online delivery modes including websites, email service, and forums to support them in practicing EBM in their rural practice.

“Webpage with frequent answers would be useful. If it’s in an email, we’ll read. If it’s not in the email then we wouldn’t know what’s the update. We wouldn’t know when it’s going to be updated. Well, in a way that we can answer the questions and then you reply us with the answer and then you attached the published paper as an extra information.”(P8 FGD 1, 31-year-old man)

“Forum. Everyone can give their own opinion, so those who have created the website can give positive feedback, so other doctors they can join in to give the opinion …” (P7 FGD 1, 31-year-old woman)

## Discussion

The key findings from this study show the complexity and challenges of practicing EBM in a rural setting. In the discussion, we will first fit the findings to the theoretical framework. Then we will discuss the five main themes found from the study namely that (1) doctors viewed evidence-based medicine mainly as statistics, research and guidelines, (2) reactions to evidence-based medicine were largely negative, (3) doctors relied on specialists, peers, guidelines and non-evidence based internet sources for information, (4) information sources were accessed using novel methods such as mobile applications and (5) there are several barriers to evidence-based practice, including doctor-, evidence-based medicine-, patient- and system-related factors.

We applied the findings to the theoretical framework as shown in Fig 1. The combination of three factors—attitude, subjective norms, and perceived behavioral control—shows that they were not practicing EBM in their clinical decision making and most had no intention to practice EBM. It is not surprising that doctors were not using evidence in their clinical practice as the theory shows that all the three domains were not supportive of influencing doctors’ behavior toward the use of EBM. The only supportive factor is that EBM was seen to be important, but this was outweighed by the barriers. In order to change behavior, one would need to reduce the main barriers by simplifying the practice of EBM, increasing awareness and knowledge of doctors and patients and improving the access to evidence.

The first theme we found was that doctors viewed evidence-based medicine mainly as statistics, research and guidelines. The definition of EBM is based on the three pillars; individual clinical expertise, patient values and expectation, and the best external evidence. Participants defined EBM mainly as research evidence and they also considered clinical practice in the definition. However, none of the interviewed doctors mentioned ‘patient values’ as a component of EBM. Patient values are defined as “the unique preferences, concerns and expectations each patient brings to a clinical encounter and which must be integrated into clinical decisions if they are to serve the patient” [22]. This glaring omission highlights the lack of awareness and attention toward patient values, which forms a key element of EBM. Patient values are also central to patient centered care. Gerteis [23] noted that one of the primary dimensions of patient centered care is respect for patients’ values, preferences, and expressed needs. Doctors must develop their skills to balance and integrate these factors, dealing not only with previous experience, but also include pertinent research evidence and patients’ preferences.

Second, doctors expressed mainly negative attitudes toward the implementation of EBM. Awareness of EBM was prevalent but it was seen as unimportant and irrelevant. Participants in this study perceived EBM as creating more workload and their work setting as lacking in resources. The most recent study conducted in the United Kingdom found that doctors were generally positive toward the practice of EBM and their knowledge about EBM was higher compared to the previous published study [24,25]. In a study of doctors in the United States, 94% agreed or strongly agreed that they were motivated to use clinical practice guidelines by a desire to improve the quality of care [26]. In the Middle East, e.g. in Oman, Bahrain, and Saudi Arabia, EBM awareness goes back to at least 1999 when pioneers introduced the concept through lectured courses [27,28]. A study in Kuwait found that EBM awareness in primary care physicians was low and training was recommended to improve practice [29]. In Bahrain, family physicians, especially those with prior EBM training, were found to be practicing EBM [30].

The third and fourth theme are related in that participants still preferred obtaining information from their peers and interestingly, the study participants used WhatsApp—a smart phone messenger—as a means of doing so. Searching information other than from peers was limited to accessing informal websites by Google search such as Wikipedia. The rapid rise in the use of smart phones has enabled advanced mobile communication between healthcare professionals. Smart phones provide access to evidence-based medical resources including disease diagnosis guides, drug references, literature search, and continuing medical education materials at the point of care [31]. Real-time clinical information is important in the practice of EBM, since clinicians may not seek answers to clinical questions after completion of a clinical encounter [32,33]. Yet, this study found that the use of smart phones did not facilitate better evidence retrieval even though it allowed availability of access to online databases. Even with a mini computer in their hands, participants still prefer the use of traditional information sources.

Finally, the barriers identified in our study were similar to those published previously such as insufficient time to access the sources, insufficient basic EBM skills, lack of time, lack of resources, lack of EBM training workshops and courses [6]. However despite advances in urban developed settings, barriers to implement EBM in resource-poor areas such as rural settings remain difficult to overcome. A study conducted among hospital practitioners in Malaysia found poor IT support at the point of care. There was a shortage of computers with reliable internet access as well as lack of time and poor awareness of EBM [34,35]. Limited resources had also been highlighted as barriers in other resource-poor settings. A study conducted in India [36] found that acceptance of EBM was not enough to support practice if facilities for searching answers to clinical questions were not available. IT resources required for finding and using research were often not available in resource-poor settings [37–39]. Participants did not have enough knowledge and skills to implement EBM in their daily clinical practice and recommended EBM training as a means to overcome this barrier.

Although there had been a number of studies conducted in which many of the barriers toward EBM were similar, this study highlights that progress was still limited in the rural setting in Malaysia. Attitudes and knowledge have remained poor despite the fact that EBM had been introduced for more than two decades. This exploratory method using a qualitative design allowed factors unique to the setting to be identified and understood. Until unique barriers were made known, interventions to tackle the challenges would not be successful. The barriers and needs identified in this study would have particular significance and value to settings with limited resources.

### Strengths and Limitations

This research highlighted the views and experiences of the doctors working in rural areas where the setting imposed different challenges from the better studied urban settings. The qualitative design allowed a multi-dimensional perspective of the data obtained from its natural setting. We believe that this explorative approach will better inform the development of interventions to overcome the barriers to the practice of EBM.

This study had some limitations. There are areas in Malaysia that are even more remote and rural where there may be greater barriers and challenges. This study was originally planned to be conducted in rural areas in Sabah in East Malaysia where health inequity issues are a problem. Due to the distance and greater resource requirements, it was decided to conduct the interviews in the state of Pahang, Peninsular Malaysia. However, it is unlikely that findings would be significantly different in the state of Sabah, as the doctors in both states work under the same healthcare system.

In this study, the researchers’ personal views and experiences might have had an influence on data analysis. NCJ and LSM are academic primary care physicians and advocates of evidence-based medicine. To minimize this bias, data were transcribed verbatim, coded and analyzed by RH, NAH and HGJ. The results were then checked and counterchecked with all members.

### Recommendations for Future Research and Clinical Practice

The barriers affecting the EBM implementation identified in this study should be targeted for intervention. This can be researched through interventional trials. We also recommend that this study be replicated in regional urban settings to investigate whether the doctors’ views and barriers are different. In clinical practice, skills for EBM can be enhanced through carefully targeted, multidisciplinary undergraduate and continuing education programs, especially for rural doctors. Evidence-based guidelines need to be locally adapted to attend to the context of rural practice. Doctors should be encouraged to involve patient values in their practice. The implementation of EBM is likely to be more successful if it is practised in ways that doctors are using currently.

## Conclusion

Most primary care physicians in rural health clinics in Malaysia do not use EBM. Factors associated with the non-use of evidence can be categorized into doctors’ barriers, EBM barriers, patient barriers and system barriers. This included lack of knowledge about EBM, attitudes and intentions toward the implementation, lack of support and encouragement from the management, lack of time to practice EBM, and insufficient access to relevant information sources. Further research is recommended to develop and evaluate interventions to overcome these identified barriers.

## Supporting Information

S1 Appendix Topic guide. (DOCX)
