# Supplementary material for: Rural Doctors’ Views on and Experiences with Evidence-Based Medicine: The FrEEDoM Qualitative Study
Source: PLoS One. 2016 Mar 31;11(3):e0152649. doi: 10.1371/journal.pone.0152649 (PMC4816333; doi:10.1371/journal.pone.0152649)
Supplement: S1 Appendix — (DOCX) [file pone.0152649.s001.docx]

## S1 Appendix. Supplementary InformationTopic Guide

**Preamble**:

1. Ice breaking/Refreshment
2. Introduction
3. Explain briefly about the objectives and method of the study
4. Distribute the Participant Information Sheet (Clarify any queries)
5. Explain:
   - No right or wrong answer
   - It is their personal views and experiences they we value
   - Can stop any time
   - Do not have to answer if they feel uncomfortable
   - Ensure confidentiality (individual and group)
   - How the data will be used
6. Explain the need to audio-record
7. Obtain written consent for the interview and audio-recording
8. Complete the participant background questionnaire

**Start the interview** (switch on the recorder)

**Questions**

- What is your view about evidence-based medicine (EBM)?
- What do you understand by EBM?
- Where did you learn about EBM?
- Do you practice EBM?
  - If yes,
    - How do you practice EBM?
    - Under what circumstances?
    - How much time do you spend?
  - If not, why not? Do you intend to practise EBM?
- How do you usually search for medical information? (Search strategy)
  - Where do you search? (e.g. CPGs, journals, books, etc) Why?
  - How do you search?
  - Do you find it easy to search for information?
    - If not, what are the reasons (barriers*)?
    - If yes, what are the reasons (facilitators*)
- Do you assess the information? (Appraisal)
  - If yes, how?
  - If no, why not?
  - Do you find it easy to assess the information?
    - If not, what are the reasons (barriers*)?
    - If yes, what are the reasons (facilitators*)
- When do you use the information? (Implementation)
  - Probe: What condition? With who?
  - Do you find it easy to use the information?
    - If not, what are the reasons (barriers*)?
    - If yes, what are the reasons (facilitators*)
- Do you have any suggestions how to make the practice of EBM easier for you?
- We are going to develop an EBM support service to provide clinical evidence to help answer your clinical queries. (Explain the intervention)
  - What do you think?
  - Explore whether it is feasible and acceptable.
- Do you have any other comments or suggestions?

**Thank you.**

* Barriers and facilitators: Probe for doctor, patient and system factors
